# Comparing diabetes prediction based on metabolic dysfunction-associated steatotic liver disease and nonalcoholic fatty liver disease: the ELSA-Brasil study

**DOI:** 10.1590/0102-311XEN009924

**Published:** 2024-11-25

**Authors:** Gabriela Wünsch Lopes, Scheine Leite Canhada, Rodrigo Citton Padilha dos Reis, Maria de Fátima Haueisen Sander Diniz, Alessandra Carvalho Goulart, Luciana Costa Faria, Rosane Harter Griep, Hugo Perazzo, Bruce Bartholow Duncan, Maria Inês Schmidt

**Affiliations:** 1 Programa de Pós-graduação em Epidemiologia, Universidade Federal do Rio Grande do Sul, Porto Alegre, Brasil.; 2 Departamento de Estatística, Universidade Federal do Rio Grande do Sul, Porto Alegre, Brasil.; 3 Faculdade de Medicina, Universidade Federal de Minas Gerais, Belo Horizonte, Brasil.; 4 Hospital das Clínicas da Faculdade de Medicina da Universidade de São Paulo, São Paulo, Brasil.; 5 Faculdade de Saúde Pública, Universidade de São Paulo, São Paulo, Brasil.; 6 Instituto Oswaldo Cruz, Fundação Oswaldo Cruz, Rio de Janeiro, Brasil.; 7 Instituto Nacional de Infectologia Evandro Chagas, Fundação Oswaldo Cruz, Rio de Janeiro, Brasil.; 8 Hospital de Clínicas de Porto Alegre, Universidade Federal do Rio Grande do Sul, Porto Alegre, Brasil.

**Keywords:** Non-Alcoholic Fatty Liver Disease, Type 2 Diabetes Mellitus, Ethnicity, Hepatopatia Gordurosa não Alcoólica, Diabetes Mellitus Tipo 2, Etnicidade, Enfermedad del Hígado Graso no Alcohólico, Diabetes Mellitus Tipo 2, Etnicidad

## Abstract

We aimed to compare nonalcoholic fatty liver disease (NAFLD) and metabolic dysfunction-associated steatotic liver disease (MASLD) definitions concerning diabetes prediction in a large sample of Brazilian adults. As a secondary objective, we compared associations between NAFLD/MASLD and diabetes across self-declared race/skin color groups. The *Brazilian Longitudinal Study of Adult Health* (ELSA-Brasil) is a prospective cohort study of Brazilian civil servants (35-74 years) enrolled from 2008 to 2010 and followed up from 2012-2014 and 2017-2019. We ascertained type 2 diabetes mellitus at baseline as well as follow-up visits based on self-reported diagnosis, medication use, and glycemic tests (fasting and 2h post-OGTT glucose and HbA1c). We excluded individuals with heavy alcohol consumption or self-reported cirrhosis/hepatitis. We analyzed 7,073 subjects. NAFLD was defined by ultrasound-based steatosis. Participants with steatosis and at least one cardiometabolic factor were considered as having MASLD. Cox proportional hazards models were performed to evaluate the association between NAFLD/MASLD and the incidence of type 2 diabetes mellitus. At baseline, 33.9% of individuals presented NAFLD and 32.5% presented MASLD. Over 9.4 years of follow-up, the relative increase in the incidence of diabetes was 78% for NAFLD (HR = 1.78; 95%CI: 1.58-2.01) and 88% for MASLD (HR = 1.88; 95%CI: 1.67-2.12). Associations did not differ significantly among race/skin color groups (p for interaction = 0.10 for MASLD and 0.08 for NAFLD). In this large cohort of middle-aged and older Brazilian adults, the relative incidence of diabetes was similar for NAFLD and MASLD definitions, with similar associations in all ethnic groups.

## Resumo

Este estudo comparou as definições de doença hepática gordurosa não alcoólica (DHGNA) e doença hepática esteatótica associada à disfunção metabólica (DHEADM) em relação à predição de diabetes em uma grande população adulta de brasileiros. Como objetivo secundário, comparamos as associações entre DHGNA/DHEADM e diabetes em grupos de diferentes etnias/cor da pele autodeclaradas. O *Estudo Longitudinal de Saúde do Adulto* (ELSA-Brasil) é um estudo de coorte prospectivo de servidores públicos brasileiros (35-74 anos) recrutados de 2008 a 2010 e acompanhados nos períodos de 2012-2014 e 2017-2019. Determinamos o diabetes mellitus tipo 2 nas visitas iniciais e de acompanhamento com base em diagnóstico autorreferido, uso de medicação e testes glicêmicos (glicemia em jejum e 2 horas após o teste de tolerância à glicose oral e HbA1c). Foram excluídos indivíduos com consumo pesado de álcool ou cirrose/hepatite autorreferida. Foram analisados 7.073 indivíduos. A DHGNA foi definida por esteatose através de ultrassom. Participantes com esteatose e pelo menos um fator cardiometabólico foram considerados como portadores de DHEADM. Modelos de riscos proporcionais de Cox foram aplicados para avaliar a associação entre DHGNA/DHEADM e a incidência de diabetes mellitus tipo 2. No início do estudo, 33,9% apresentavam DHGNA e 32,5% DHEADM. Ao longo de 9,4 anos de acompanhamento, o aumento relativo na incidência de diabetes foi de 78% para indivíduos com DHGNA (HR = 1,78; IC95%: 1,58-2,01) e 88% para indivíduos com DHEADM (HR = 1,88; IC95%: 1,67-2,12). As associações não diferiram significativamente entre os grupos de etnia/cor de pele (p para interação = 0,10 para DHEADM e 0,08 para DHGNA). Nesta grande coorte de brasileiros de meia-idade e idosos, a incidência relativa de diabetes foi semelhante nas definições de DHGNA e DHEADM, com associações semelhantes entre grupos étnicos.

## Resumen

Este estudio comparó las definiciones de enfermedad del hígado graso no alcohólico (EHGNA) y enfermedad hepática esteatótica asociada con disfunción metabólica (EHDM) con relación a la predicción de diabetes en una gran población adulta de brasileños. Como objetivo secundario, comparamos las asociaciones entre EHGNA/MASLD y diabetes en grupos de diferentes etnias/color de piel autodeclarados. El *Estudio Longitudinal de Salud del Adulto* (ELSA-Brasil) es un estudio de cohorte prospectivo de servidores públicos brasileños (35-74 años) reclutados entre el 2008 y el 2010 y seguidos en los períodos 2012-2014 y 2017-2019. Determinamos la diabetes mellitus tipo 2 en las visitas iniciales y de seguimiento con base en el diagnóstico autoinformado, el uso de medicación y las pruebas de glucemia (glucemia en ayunas y prueba de tolerancia a la glucosa oral 2 horas después y HbA1c). Se excluyeron las personas con consumo excesivo de alcohol o cirrosis/hepatitis autoinformadas. Se analizaron 7.073 individuos. La EHGNA se definió por esteatosis mediante ecografía. Se consideró que los participantes con esteatosis y al menos un factor cardiometabólico tenían EHDM. Se aplicaron modelos de riesgos proporcionales de Cox para evaluar la asociación entre EHGNA/EHDM y la incidencia de diabetes mellitus tipo 2. Al inicio del estudio, el 33,9% tenía EHGNA y el 32,5% EHDM. Durante 9,4 años de seguimiento, el aumento relativo en la incidencia de diabetes fue del 78% para las personas con EHGNA (HR = 1,78; IC95%: 1,58-2,01) y del 88% para las personas con EHDM (HR = 1,88; IC95%: 1,67-2,12). Las asociaciones no difirieron significativamente entre los grupos étnicos/color de piel (p para interacción = 0,10 para EHDM y 0,08 para EHGNA). En esta gran cohorte de brasileños de mediana edad y ancianos, la incidencia relativa de diabetes fue similar en las definiciones de EHGNA y EHDM, con asociaciones similares entre grupos étnicos.

## Introduction

The burden of type 2 diabetes mellitus and nonalcoholic fatty liver disease (NAFLD) has increased in the last decades. From 1990 to 2019, the global incidence of NAFLD increased by 95%, and deaths and disability-adjusted life years (DALYs) attributable to NAFLD increased by 80% and 63%, respectively ^1^. In the same period, new cases of type 2 diabetes mellitus increased by 78%, type 2 diabetes mellitus-related deaths by 68%, and DALYs by 80% worldwide ^2^.

Positive associations between NAFLD and incident type 2 diabetes have been well documented ^3^, and their causal nature was demonstrated by Mendelian randomization ^4^. Consistent with these findings, we also found a higher risk of diabetes associated with NAFLD ^5^ in Brazilian adults based on an early follow-up examination of the *Brazilian Longitudinal Study of Adult Health* (ELSA-Brasil).

In 2023, a new definition and criteria for steatotic liver disease emerged to replace the nomenclature used in previous definitions. Proposed by a consensus of specialists, “metabolic dysfunction-associated steatotic liver disease” (MASLD) ^6^ excludes other causes of steatosis but requires the presence of metabolic dysfunction. To our knowledge, the only study contrasting MASLD with the previous NAFLD definition regarding diabetes prediction was done in a Chinese sample ^7^.

Ethnic disparities related to NAFLD occurrence and complications have been reported, with a lower prevalence of NAFLD being found in black individuals ^8^ ^,^ ^9^. A North American cohort ^10^ showed an increased absolute and relative risk of diabetes related to NAFLD in white but not in black participants, which is surprising considering the higher burden of diabetes experienced by black individuals ^11^ ^,^ ^12^.

To provide additional information regarding these issues, our objectives were to: (1) reassess the association of NAFLD with the incidence of type 2 diabetes mellitus and contrast it with the association found with the new MASLD definition; and (2) compare associations between NAFLD/MASLD and diabetes among self-declared Brazilian ethnic groups.

## Methods

### Study population and design

The ELSA-Brasil is an ongoing prospective multicenter cohort study of civil servants aimed at investigating risk factors for the development of chronic diseases such as diabetes ^13^. During the baseline visit, the study enrolled 15,105 participants aged 35-74 years at public institutions in six capitals of Brazilian states (Bahia, Espírito Santo, Minas Gerais, Rio de Janeiro, Rio Grande do Sul, and São Paulo) from 2008 to 2010 ^14^. Two follow-up visits occurred between 2012-2014 and 2017-2019. Ethics committees of each institution involved in the study approved the research protocol, and participants signed informed consent forms agreeing to participate in each visit ^15^.

Participants underwent standardized questionnaires, abdominal ultrasound, and blood sample collection after an overnight fasting ^16^ with strict quality control ^17^. Blood samples were analyzed in a centralized laboratory ^18^. In addition to clinical visits, participants responded to annual telephone questions, including whether and when a new medical diagnosis of diabetes was made since the last visit ^19^.

**Figure 1:**
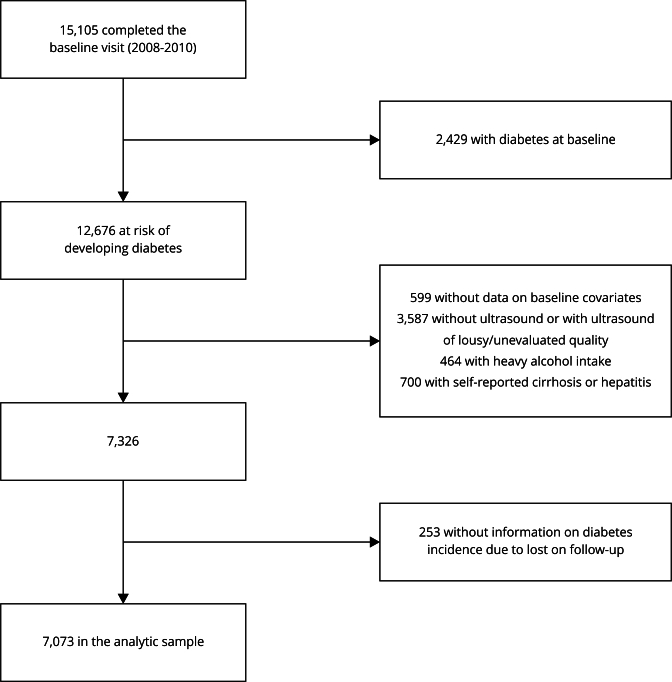
Flowchart of analytic sample selection for the assessment of incident diabetes.

In this analysis, 2,429 (16%) participants with prevalent diabetes were excluded, as well as 599 individuals without data on baseline covariates, 2,295 subjects who did not undergo baseline ultrasonography, and 1,292 participants who did not have information on ultrasonography quality or had a ultrasonography quality classified as “poor” or “unacceptable” according to liver ultrasound parameters used for the diagnosis of steatosis (see ahead). To follow NAFLD ^20^ and MASLD ^6^ definitions, 464 individuals with heavy alcohol intake at baseline (> 140g of alcohol per week for females and > 210g of alcohol per week for males) were excluded in addition to other 700 who reported a medical diagnosis of cirrhosis or hepatitis at baseline. We also excluded 253 participants without information on diabetes incidence due to loss on follow-up. Finally, data from 7,073 participants were analyzed (Figure 1).

### Assessment of diabetes

Prevalent diabetes was defined by (1) self-report (answering “Yes” to the question “Have you been previously told by a physician that you had/have diabetes?”; (2) medication use (answering “Yes” to the question “Have you used medication for diabetes or high blood sugar in the past two weeks?”; or (3) abnormal glycemic tests: fasting plasma glucose (FPG; ≥ 126mg/dL, ≥ 7mmol/L), 2-hour oral glucose tolerance test (2h-75g OGTT; 200mg/dL, ≥ 11.1mmol/L), and glycated hemoglobin (HbA1c; ≥ 6.5%, ≥ 48mmol/mol) ^21^ ^,^ ^22^.

Also new cases of diabetes developed during follow-up were identified among those free of the disease at baseline, using information gathered during follow-up visits (questionnaires and laboratory tests) or the annual telephone surveillance. The time until the development of diabetes or censure was estimated as previously reported ^23^.

### Assessment of NAFLD and MASLD

Steatosis by liver ultrasonography was assessed only at baseline ^16^ ^,^ ^24^ using the same equipment model in all research centers: a high-resolution B-mode scanner (SSA-790A, Aplio XG, Toshiba Medical Systems; https://www.global.toshiba) with a convex array transducer (model PVT-375BT) adjusted with 3.5 MHz central frequency and fundamental frequency ranging 1.9-5.0 MHz. The exam was carried out by board-certified radiologists or trained technicians following a standardized protocol. The parameters evaluated were hepatic beam attenuation, anteroposterior diameter of the right hepatic lobe, and hepatorenal index. A centralized reading center in São Paulo interpreted the exams, and a senior radiologist evaluated their quality.

Steatosis was defined based on hepatic beam attenuation, our study’s most accurate measure for hepatic steatosis when compared with high-resolution computerized tomography in participants with NAFLD ^24^. Hepatic beam attenuation was based on the visibility of intra-hepatic vessels and the diaphragm posterior to the right hepatic lobe. The absence of steatosis was defined by a normal hepatic attenuation with complete diaphragm visualization. Abnormal attenuation was classified into mild (diaphragm > 50% visible), moderate (diaphragm < 50% visible), or severe (no visible diaphragm) ^24^. The ultrasonography quality was based on identifying four anatomic marks (anterior hepatic surface, posterior hepatic surface, gallbladder, and portal vein) in images of the right hepatic lobe in the axial plane. Exams in which fewer than two anatomical landmarks were identified were considered “poor”.

NAFLD was defined by ultrasound-based steatosis at baseline. MASLD was defined by ultrasound-based steatosis and the presence of at least one of the following cardiometabolic risk factors, according to the MASLD definition: overweight/obesity defined by elevated body mass index (BMI) or elevated waist circumference (WC), prediabetes, elevated blood pressure, hypertriglyceridemia, and low HDL-cholesterol ^6^.

Height was measured with a stadiometer and weight with an electronic scale. WC was measured using an inelastic tape at the midpoint between the lower rib margin and the anterior superior iliac crest in the mid-axillary line. BMI was calculated as weight (kg) divided by height squared (m^2^). Overweight/obesity as a cardiometabolic criterion for MASLD was defined as BMI ≥ 23kg/m^2^ for self-declared Asian and a BMI ≥ 25kg/m^2^ for other self-declared ethnicities groups, or WC > 94cm for males and > 80cm for females ^6^.

Prediabetes was defined by blood tests performed in each visit: FPG ≥ 100mg/dL (5.6mmol/L), 2h-75g OGTT ≥ 140mg/dL (7.8mmol/L), or HbA1c ≥ 5.7% (39mmol/mol) ^6^.

Blood pressure was measured trice during the clinic visits, and the mean of the last two measurements was calculated. Use of anti-hypertensive medication was defined as the presence of both: (1) the self-reported use of medication to treat arterial hypertension in the previous two weeks and (2) confirmation of an anti-hypertensive drug by cross-checking prescriptions and boxes brought by the participants. Elevated blood pressure as a MASLD criterion ^6^ was defined as a mean systolic pressure ≥ 130mmHg and/or a mean diastolic pressure ≥ 85mmHg, or by use of anti-hypertensive medication.

Hypertriglyceridemia as a MASLD criterion ^6^ was defined as plasma triglycerides ≥ 150mg/dL (1.7mmol/L) in fasting blood collection or using a triglyceride-lowering medication (fibrates or nicotinic acid) checked in prescriptions and boxes brought by the participant ^18^.

Low HDL-cholesterol as a MASLD criterion ^6^ was defined as plasma HDL-cholesterol ≤ 40mg/dL (1.0mmol/L) in males or ≤ 50mg/dL (1.3mmol/L) in females in fasting blood collection, or by use of a lipid-lowering medication (same as in hypertriglyceridemia).

### Baseline covariates

During interviews, data were collected on: age (in years), sex (female/male), self-reported skin color/race (Brazilian census categories of white, black, brown [mixed-race], Asian, and Indigenous), education (less than high school, high school, university degree), per capita household income (in Brazilian Real - BRL), history of diabetes in first-degree relatives (yes/no), and smoking status (current, former, or never).

Information about alcohol consumption (g/week) was obtained by a questionnaire considering the frequency and quantity of beer (considered as having 5% alcohol), wine, and distillates consumption. Participants were categorized into abstainers (0g/week) and light to moderate (1-140g/week for females and 1-210g/week for males) drinkers. The long form of the *International Physical Activity Questionnaire* (IPAQ) for leisure and transportation domains ^25^, validated for the Brazilian population ^26^ ^,^ ^27^, was used to estimate the weekly volume of physical activity in metabolic equivalents (METs) according to instrument guidelines.

### Statistical analysis

Continuous normal variables were presented as means and standard deviation, and continuous non-normal variables as medians and interquartile intervals. Normality was verified with the Shapiro-Wilk test. Categorical variables were described as frequencies and percentages. The Cox proportional hazards model was used to estimate hazard ratios (HR) and 95% confidence intervals (95%CI) for diabetes. The proportional hazards assumption was assured with the analysis of Schoenfeld residuals.

The prevalence of hypertension was described at baseline only for description purposes, since our exposure variable (MASLD) also contains a measure of high blood pressure. In Table 1, the variable “Hypertension” was defined as confirmed use of anti-hypertensive medications in the previous two weeks or mean high blood pressure (≥ 140 and/or ≥ 90 mmHg) measured at the clinic ^28^.

**Table 1:** Baseline characteristics in the overall sample and according to nonalcoholic fatty liver disease (NAFLD) and metabolic dysfunction-associated steatotic liver disease (MASLD) definitions.

Characteristics	Total [N = 7,073]	NAFLD [N = 2,395]	MASLD [N = 2,298]
	n (%)	n (%)	n (%)
Age (years) [mean (SD)]	50.8 (8.87)	51.5 (8.68)	51.6 (8.67)
Sex: female	4,202 (59.4)	1,229 (51.3)	1,174 (51.1)
Self-identified race/skin color			
White	4,008 (56.7)	1,370 (57.2)	1,303 (56.7)
Mixed-race	1,778 (25.1)	606 (25.3)	585 (25.5)
Black	1,036 (14.6)	343 (14.3)	335 (14.6)
Asian	187 (2.7)	52 (2.2)	52 (2.2)
Indigenous	64 (0.9)	24 (1.0)	23 (1.0)
Education			
Less than high school	755 (10.7)	302 (12.6)	296 (12.9)
High school	2,530 (35.8)	893 (37.3)	871 (37.9)
University degree	3,788 (53.6)	1,200 (50.1)	1,131 (49.2)
Family history of diabetes	2,584 (36.5)	927 (38.7)	897 (39.0)
Hypertension	1,996 (28.2)	886 (37.0)	886 (38.6)
Alcohol consumption categories			
Abstemious	3,881 (54.9)	1,269 (53.0)	1,225 (53.3)
Light-moderate	3,192 (45.1)	1,126 (47.0)	1,073 (46.7)
Smoking status			
Current	851 (12.0)	280 (11.7)	270 (11.7)
Former	1,988 (28.1)	759 (31.7)	735 (32.0)
Never	4,234 (59.9)	1,356 (56.6)	1,293 (56.3)

SD: standard deviation.

For each predictor (NAFLD and MASLD) two models were performed. Model 1 was unadjusted and Model 2 included age (years), sex (male/female), study center (Bahia, Espírito Santo, Minas Gerais, Rio de Janeiro, Rio Grande do Sul, São Paulo), race/skin color (white, mixed-race, black, others), education (less than high school, high school, university degree), per capita household income (BRL), smoking status (current, former, never), alcohol intake categories (abstinence, light-moderate drinking), physical activity (METs/week), and family history of diabetes mellitus (yes/no). The variables for adjustment were selected based on previous literature evaluating diabetes risk ^29^. These variables are generally also related to MASLD. Variables that were part of the MASLD definition were not included in the model adjustment. Variance inflation factors were used to assess important collinearity (> 5).

Additional models were performed, including interaction terms for NAFLD and MASLD with race/skin color groups. For interaction analyses, subjects who self-declared as Asian or Indigenous were grouped in the category “Other”, due to small numbers. However, black and brown/mixed-race individuals were not grouped to permit assessment of a graded increased risk of diabetes among all groups. Black and mixed-race subjects are at increased risk of diabetes, and they are unfavorably exposed to the social determinants of health compared to white subjects ^30^. Thus, tests were performed for effect modification by race/skin color categories using the likelihood ratio test, comparing the goodness of fit between the models with and without the interaction terms.

All the analyses were performed using software R, version 4.3.0 (http://www.r-project.org). A 2-sided p-value < 0.05 was considered statistically significant for all associations.

## Results

Of the 7,073 individuals in the analytic sample free of diabetes at baseline, 2,395 participants (33.9%) were classified as having NAFLD and 2,298 (32.5%) had MASLD. Most subjects with NAFLD (96%) were also classified as having MASLD.

Our sample comprised males and females with an average age of 51 years and of various self-declared multiethnic groups, including 1,036 (14.6%) individuals self-declared as black and 1,778 (25.1%) as mixed-race. Less than 4% individuals self-declared as Asian or Indigenous. Almost half did not have a university degree. Family history of diabetes and diagnosis of hypertension were frequent, and current smoking was uncommon (Table 1).

Those with NAFLD or MASLD at baseline were similar regarding these characteristics in all ethnic groups. The most common cardiometabolic risk factor among those with MASLD was overweight/obesity (BMI ≥ 25kg/m^2^ or BMI ≥ 23kg/m^2^ in Asians) in all ethnic groups. Compared to white and mixed-race subjects, black individuals with MASLD presented a higher prevalence of the following metabolic abnormalities: elevated BMI, elevated WC, elevated blood pressure or medication, prediabetes, and hypertriglyceridemia, but a smaller prevalence of low HDL (Tables 2, 3, and 4).

**Table 2:** Prevalence of cardiometabolic abnormalities defining metabolic dysfunction-associated steatotic liver disease (MASLD) in subjects with nonalcoholic fatty liver disease (NAFLD) and MASLD.

Abnormalities	NAFLD [N = 2,395]	MASLD [N = 2,298]
	n (%)	n (%)
BMI ≥ 25kg/m^2^ (Asian: ≥ 23kg/m^2^)	1,894 (79.1)	1,894 (82.5)
Elevated waist circumference (> 94cm men, > 80cm women)	1,784 (74.5)	1,784 (77.6)
Blood pressure ≥ 130/85mmHg or use of anti-hypertensives	1,148 (48.0)	1,148 (50.0)
Prediabetes *	1,574 (66.1)	1,574 (68.9)
Low plasma HDL cholesterol ** or lipid-lowering medication	745 (31.2)	745 (32.5)
Plasma triglycerides ≥ 1.70mmol/L or lipid-lowering medication	1,580 (66.1)	1,483 (64.7)

BMI: body mass index.

* Since we excluded prevalent type 2 diabetes, this glucose criterion included only subjects with pre-diabetes, defined by fasting serum glucose ≥ 5.6mmol/L, 2-h OGTT ≥ 7.8mmol/L, or HbA1c ≥ 39mmol/L;

** ≤ 1.00mmol/L for men; ≤ 1.3mmol/L for women.

**Table 3:** Prevalence of cardiometabolic abnormalities defining metabolic dysfunction-associated steatotic liver disease (MASLD) in subjects with nonalcoholic fatty liver disease (NAFLD) according to self-declared race/color groups.

Abnormalities	Total [N = 2,395]	White [N = 1,370]	Mixed-race [N = 606]	Black [N = 343]	Asian [N = 52]	Indigenous [N = 24]
n (%)	n (%)	n (%)	n (%)	n (%)	n (%)
BMI ≥ 25kg/m^2^ (Asian: ≥ 23kg/m^2^)	1,894 (79.1)	1,062 (77.5)	490 (80.9)	278 (81.3)	45 (86.5)	19 (79.2)
Elevated waist circumference (> 94cm men, > 80cm women)	1,784 (74.5)	1,017 (74.2)	448 (73.9)	273 (79.6)	29 (55.8)	17 (70.8)
Blood pressure ≥ 130/85mmHg or use of anti-hypertensives	1,148 (48.0)	606 (44.3)	303 (50.0)	205 (59.8)	26 (50.0)	8 (33.3)
Prediabetes *	1,574 (66.1)	890 (65.1)	393 (65.4)	236 (69.4)	41 (80.4)	14 (58.3)
Low plasma HDL cholesterol ** or lipid-lowering medication	745 (31.2)	416 (30.4)	213 (35.2)	97 (28.3)	13 (25.0)	6 (25.0)
Plasma triglycerides ≥ 1.70mmol/L or lipid-lowering medication	1,580 (66.1)	871 (63.8)	387 (64.0)	270 (78.7)	37 (71.2)	15 (62.5)

BMI: body mass index.

* Since we excluded prevalent type 2 diabetes, this glucose criterion included only subjects with pre-diabetes, defined by fasting serum glucose ≥ 5.6mmol/L, 2h OGTT ≥ 7.8mmol/L, or HbA1c ≥ 39mmol/L;

** ≤ 1.00mmol/L for men; ≤ 1.3mmol/L for women.

**Table 4:** Prevalence of cardiometabolic abnormalities defining metabolic dysfunction-associated steatotic liver disease (MASLD) in subjects with MASLD according to self-declared race/color groups.

Abnormalities	Total [N = 2,298]	White [N = 1,303]	Mixed-race [N = 585]	Black [N = 335]	Asian [N = 52]	Indigenous [N = 23]
	n (%)	n (%)	n (%)	n (%)	n (%)	n (%)
BMI ≥ 25kg/m^2^ (Asian: ≥ 23kg/m^2^)	1,894 (82.5)	1,062 (81.5)	490 (83.8)	278 (83.2)	45 (86.5)	19 (82.6)
Elevated waist circumference (> 94cm men, > 80cm women)	1,784 (77.6)	1,017 (78.1)	448 (76.6)	273 (81.5)	29 (55.8)	17 (73.9)
Blood pressure ≥ 130/85mmHg or use of anti-hypertensives	1,148 (50.0)	606 (46.6)	303 (51.8)	205 (61.2)	26 (50.0)	8 (34.8)
Prediabetes *	1,574 (68.9)	890 (68.5)	393 (67.8)	236 (71.1)	41 (80.4)	14 (60.9)
Low plasma HDL cholesterol ** or lipid-lowering medication	745 (32.5)	416 (32.0)	213 (36.5)	97 (29.0)	13 (25.0)	6 (26.1)
Plasma triglycerides ≥ 1.70mmol/L or lipid-lowering medication	1,483 (64.7)	804 (61.9)	366 (62.7)	262 (78.2)	37 (71.2)	14 (60.9)

BMI: body mass index.

* Since we excluded prevalent type 2 diabetes, this glucose criterion included only subjects with pre-diabetes, defined by fasting serum glucose ≥ 5.6mmol/L, 2h OGTT ≥ 7.8mmol/L, or HbA1c ≥ 39mmol/L;

** ≤ 1.00mmol/L for men; ≤ 1.3mmol/L for women.

### Prediction of incident diabetes from NAFLD and MASLD

A total of 1,157 (16%) new cases of type 2 diabetes mellitus were identified during a median (interquartile range - IQR) follow-up of 9.43 (8.86; 9.87) years. In unadjusted models, people with NAFLD (HR = 1.87; 95%CI: 1.67-2.10) or MASLD (HR = 1.99; 95%CI: 1.77-2.23) were at higher risk of developing type 2 diabetes mellitus. After adjustment for covariates, the association between liver disease and incidence of type 2 diabetes mellitus remained significant (NAFLD HR = 1.78; 95%CI: 1.58-2.01; MASLD HR = 1.88; 95%CI: 1.67-2.12) (Table 5).

**Table 5:** Association between baseline liver disease and diabetes: nonalcoholic fatty liver disease (NAFLD) compared to non-NAFLD, metabolic dysfunction-associated steatotic liver disease (MASLD) compared to non-MASLD.

Predictor	n	Unadjusted		Adjusted *	
		HR	95%CI	HR	95%CI
Non-MASLD	4,775	1.00		1.00	
MASLD	2,298	1.99	1.77-2.23	1.88	1.67-2.12
Non-NAFLD	4,678	1.00		1.00	
NAFLD	2,395	1.87	1.67-2.10	1.78	1.58-2.01

95%CI: 95% confidence interval; HR: hazard ratio.

* Adjusted for age, sex, study center, race/skin color, education, per capita household income (BRL), smoking status, alcohol intake, physical activity, and family history of diabetes.

### Heterogeneity among race/skin color groups

**Figure 2:**
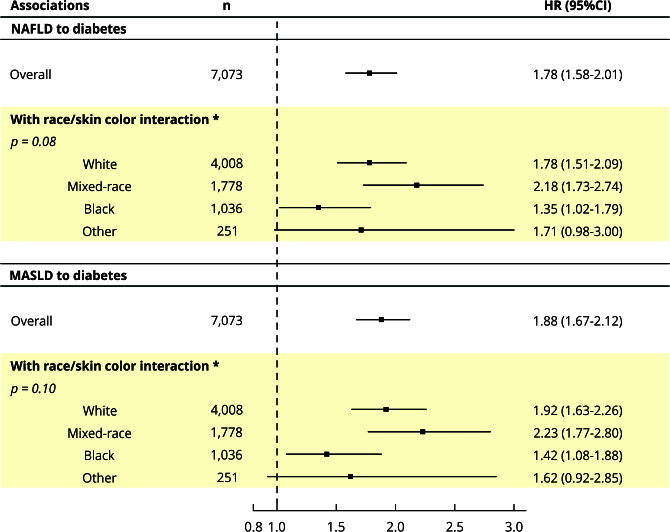
Associations between nonalcoholic fatty liver disease (NAFLD) and metabolic dysfunction-associated steatotic liver disease (MASLD) with incident diabetes according to race/color groups.

We did not find statistically significant heterogeneity in the associations among race/skin color groups for both definitions of steatotic liver disease (p for heterogeneity for NAFLD = 0.08; MASLD = 0.10), although p-values were close to the 5% criterion. In addition, as seen in Figure 2, white, black, and mixed-race individuals had increased diabetes risk related to steatotic liver disease, regardless of the definition used.

95%CI: 95% confidence interval; HR: hazard ratio. * Adjustment for age, sex, study center, education, per capita household income (BRL), smoking status, alcohol intake, physical activity, and family history of diabetes.

## Discussion

To the best of our knowledge, this is the first study investigating the association between MASLD and diabetes in a non-Asian sample. Our findings show that within an average of 9 years of follow-up, having MASLD at baseline predicted the development of type 2 diabetes in a similar manner as having NAFLD. Although primarily based on white participants, we had a considerable number of black and mixed-race subjects, finding a similar increased risk in all ethnic groups.

We found a major overlap between NAFLD and MASLD, consistent with a previous report ^31^ in the same sample, highlighting the fact that metabolic dysfunction plays a central role in this type of steatotic liver disease. A major overlap was also seen in a Chinese sample of individuals aged 18 years or older ^7^. However, our findings differ from those of the Chinese sample in two ways. First, our prevalence of steatosis was much higher, 34% vs. 18%. Second, the associations with diabetes in the Chinese sample were much larger for both NAFLD and MASLD. In fact, the associations between NAFLD and the incidence of diabetes found in that study are among the largest published so far ^3^ ^,^ ^32^.

Of note, obesity, which is occurring in epidemic proportions globally ^33^, was the most important component of steatotic liver disease regardless of the definition used. This highlights the importance of interventions to counter obesity in the population.

Concerning race/color, we provide new evidence complementing previous findings based on a U.S. sample that reported an increased risk of diabetes associated with NAFLD in whites but not in black individuals ^10^. Rather than the absence of an increased risk of NAFLD-related diabetes, we demonstrated that black and mixed-race subjects exposed to NAFLD or MASLD have an increased risk of diabetes compared to their non-exposed counterparts.

Explanations previously proposed for racial differences related to NAFLD were potential ethnic differences in body fat distribution ^34^, lipid profile ^34^ ^,^ ^35^, and health social determinants ^36^. The Brazilian self-declared classification of Afro-descendants into black and brown [mixed-race] individuals is a way to capture differential exposure to health social determinants related to the construct of race/skin color. Previous studies from ELSA-Brasil have shown that both black and mixed-race subjects face disproportionate exposure to adverse life conditions compared to whites, such as more residential segregation ^37^ and lower socioeconomic position ^38^. However, compared to mixed-race, black individuals experienced higher levels of racial discrimination ^39^, were more likely to live in segregated neighborhoods, and had higher adjusted prevalences of hypertension and diabetes ^37^. Despite these disparities, our findings did not show a gradient in the risk of diabetes among race groups.

Potential limitations of our findings must be considered. First, residual confounding is always possible in observational studies, although we included important variables related to diabetes and MASLD. Second, although our diabetes ascertainment was very comprehensive, based on fasting and 2h glycemia, as well as HbA1c levels, it may have high sensitivity but low specificity, perhaps introducing more false positive incident cases and thus decreasing the true magnitude of the association. Third, we did not exclude other less frequent secondary causes of steatosis and did not evaluate indicators of liver fibrosis and other advanced liver diseases due to the unavailability of this information. Fourth, our models were adjusted only by confounding variables measured at baseline, overlooking potential changes in the effect of confounders across time, which seems especially relevant for lifestyle habits such as alcohol consumption and physical activity.

Our findings have some strengths to be highlighted. Being a large prospective cohort study with highly standardized measurements and numerous relevant covariates, we were able to adjust for multiple confounders addressed in the literature ^40^ and to evaluate possible interactions among race/skin color groups. In this regard, we provided information on self-reported race/skin color in the association of incident diabetes with MASLD, supporting the use of the new MASLD definition to predict diabetes in various ethnic groups.

Finally, although the new MASLD definition provides similar information compared to the previous NAFLD in terms of diabetes prediction, it may offer additional advantages at the nomenclature level. However, additional investigation is needed. The inclusion of individuals with heavy alcohol intake and secondary causes of liver disease, now defined as metabolic dysfunction and alcohol-associated liver disease (MetALD) and MASLD with combined etiology ^6^, have not been evaluated regarding diabetes and other cardiometabolic conditions. In addition, the impact of applying ethnic-specific waist circumference cutoffs in the MASLD definition merits further exploration. Finally, further evaluation of health social determinants regarding the MASLD-diabetes association in different ethnic groups is warranted.

## Conclusion

In this Brazilian sample of self-declaring white, mixed-race, black, and in lower proportions also Asian and Indigenous subjects, MASLD predicted similar relative risks of diabetes compared to NAFLD, with similar magnitudes in all ethnic groups.
